# The quality of doctoral nursing education in South Africa

**DOI:** 10.4102/curationis.v38i1.1441

**Published:** 2015-07-09

**Authors:** Siedine K. Coetzee, Hester C. Klopper, Mi J. Kim

**Affiliations:** 1 School of Nursing Science, North-West University, Potchefstroom Campus, South Africa; 2 College of Nursing, University of Illinois at Chicago, Chicago, United States

## Abstract

**Background:**

The number of doctoral programmes in nursing has multiplied rapidly throughout the world. This has led to widespread concern about nursing doctoral education, specifically with regard to the quality of curricula and faculty, as well as to the availability of appropriate institutional resources. In South Africa, no study of these issues has been conducted at a national level.

**Objective:**

To explore and describe the quality of nursing doctoral education in South Africa from the perspectives of deans, faculty, doctoral graduates and students.

**Method:**

A cross-sectional survey design was used. All deans (*N* = 15; *n* = 12), faculty (*N* = 50; *n* = 26), doctoral graduates (*N* = 43; *n* = 26) and students (*N* = 106; *n* = 63) at South African nursing schools that offer a nursing doctoral programme (*N* = 16; *n* = 15) were invited to participate. Data were collected by means of structured email-mediated Quality of Nursing Doctoral Education surveys.

**Results:**

Overall, the graduate participants scored their programme quality most positively of all the groups and faculty scored it most negatively. All of the groups rated the quality of their doctoral programmes as good, but certain problems related to the quality of resources, students and faculty were identified.

**Conclusion:**

These evaluations, by the people directly involved in the programmes, demonstrated significant differences amongst the groups and thus provide valuable baseline data for building strategies to improve the quality of doctoral nursing education in South Africa.

## Introduction

Increasingly, the nursing profession is challenged by market demands, compelling expectations of more efficient and better quality services, as well as escalating financial pressures (Ketefian et  al. 2005; Woodford & Nyquist 2005). These make it imperative for the nursing profession to develop a relevant body of knowledge and skills that meets the changing needs and that provides leadership firmly grounded in both knowledge and high-level expertise (Slevin & Hanucharurnkul 2005). Nursing doctoral education has been identified as critical in developing scholarly leaders in practice, management, research, policy and education and training (Ketefian et  al. 2005). Since the inception of nursing doctoral education in the USA in 1954, it has multiplied to 131 research-focused programmes in the USA alone (American Association of Colleges of Nursing [AACN] 2012), with over 333 programmes in 34 countries worldwide (International Network of Doctoral Education in Nursing [INDEN] 2013).

### Background

In South Africa (SA), the first students were enrolled in a nursing doctoral programme in 1970 (Potgieter 1992); since then 16 schools of nursing offer doctoral degree courses, all of which are research intensive with no structured coursework. The programmes are guided by the Higher Education Qualifications Framework, which legislates the classification, registration, publication and quality of higher education qualifications in conjunction with the South African Qualification Authority, the Higher Education Quality Council and the Council on Higher Education (CHE 2009) regulatory bodies. These bodies provide Higher Education Institutions (HEI) with a broad indication of learning achievements or outcomes that need to be attained at doctoral level (Department of Education [DoE] 1997), but they do not prescribe the particulars of a doctoral programme. Thus, even though all 16 SA nursing schools offer research-focused doctoral degrees, each differs significantly regarding title, mode of delivery, prerequisites, content, assessment standards and awarding of nursing doctorates. This variety illustrates the fact that doctoral programmes vary, not only amongst countries, but also amongst schools of nursing within countries.

The rapid growth and the differences amongst nursing doctoral programmes worldwide have caused many leaders in the field to be concerned about the quality of nursing doctoral education (Kim et  al. 2011). This concern motivated the Quality Standards, Criteria and Indicators (QSCI) task team of INDEN to develop global QSCI for nursing doctoral education that can be used to evaluate the quality of doctoral education globally and identify threats to such quality (Kim, McKenna & Ketefian 2006). The seven major criteria identified in the QSCI investigation included: the nature of the mission; the quality of faculty; doctoral students; curriculum; programme administration; availability of institutional resources; and evaluation of the programme, with sub-criteria, standards and indicators to measure the quality of each specific criterion (Kim & Ketefian 2004; Kim et  al. 2006).

### Problem statement

Strategies are being developed globally to increase the number of faculty with doctoral qualifications, strengthen faculty research portfolios, improve resources and infrastructure to support students, increase funding for research activities of faculty and students and prepare students for the global marketplace. In SA, however, the current status of nursing doctorates is relatively unexplored and no study has been conducted nationally to evaluate the quality of nursing doctoral education. We propose that examining the perspectives of different groups of people involved in doctoral programmes in SA using global QSCI, will provide baseline data that will allow threats to quality to be identified, as well as allow a countrywide strategy to improve the quality of doctoral education in nursing in SA to be developed.

#### Aim of the study

This study aimed to compare the perspectives of deans, faculty, doctoral graduates and students on the quality of nursing doctoral education, focusing on: the nature of the mission; the quality of faculty, doctoral students, curriculum, programme administration and infrastructure; availability of institutional resources; and evaluation of the programme.

## Research design

### Research approach and method

This research forms part of an international collaborative study to compare the quality of nursing doctoral education in Australia, Japan, Korea, SA, Thailand, UK and the USA; and to develop strategies for improving the quality of doctoral nursing education amongst these countries. A descriptive, cross-sectional comparative design was used.

#### Population and sampling

All 16 nursing schools that offer a doctoral programme in nursing in SA were invited to participate in the study, but only the 15 that granted ethical permission were included. All deans, faculty, graduates and students from the 15 nursing schools were invited to participate. For the purpose of our study, a nursing school refers to a nursing school/department/division in an HEI that offers a doctoral programme in nursing. The deans were the heads of the nursing schools; faculty were employees at the nursing school who contributed to its doctoral programme; graduates were those who had completed their doctorates at a nursing school in the previous three years; and students were those registered for doctoral degrees in nursing at the time of our investigation.

#### Ethical considerations

The research study was introduced to deans at a business meeting of the Forum of University Nursing Deans in SA and each was provided with an information leaflet and a consent form. Two weeks later, enquiries were sent to the deans by email as to whether their respective nursing school could be included in the study. A month after the first request, the deans who had not responded to the first enquiry were again reminded by email. Fifteen nursing schools were included in this study after permission was granted by their dean, the HEI's ethical review boards and/or institutional management. Deans were asked to provide the name and contact information of the coordinator of the doctoral programme at the nursing school, who was then contacted and asked for a list of faculty, doctoral graduates and students with their email addresses. Each person on the list was contacted via email and asked to participate in the research study; an information leaflet and the survey were attached. Two weeks after distribution of the surveys, a second email was sent with the information leaflet and survey attached, as a reminder to non-responders. A third email with the information leaflet and survey attached was sent to non-responders a month after the first contact. Ethical approval was granted by the Institutional Ethical Review Board of the North-West University (NWU-0085-08-S5).

#### Measures

Our study used the Quality of Nursing Doctoral Education surveys that were developed from the work of the QSCI task team, whose members were charged by INDEN in 2000 to determine the standards of doctoral education in nursing worldwide (Kim & Ketefian 2004). This task team consisted of 15 members from 8 countries: Australia, Brazil, Canada, Korea, Poland, SA, UK and the USA. The QSCI document was developed on the basis of the Position Statement of the AACN, *Indicators of quality in research-focused doctoral programs in nursing* (AACN 2001). Task force members provided evaluation of the QSCI document from their countries’ perspective three times over a three-year period; this was then circulated to all INDEN members seeking their inputs. Standards, criteria or indicators that were identified by members to be inapplicable in their countries were removed in the final version of the document (Kim & Ketefian 2004).

The seven major criteria that it contained for assessing doctoral nursing programmes were: the nature of the mission; the quality of the faculty, doctoral students, curriculum and programme administration and infrastructure; the availability of institutional resources; and evaluation (Kim & Ketefian 2004; Kim et  al. 2006). From these, a set of four Quality of Nursing Doctoral Education surveys was developed for the dean, faculty, graduates and students and these were distributed to every participating nursing school. Items for the last three groups were the same; those for the deans were different. The deans’ survey comprised three sections that evaluated the quality of doctoral education: Section 1 (22 items) focused on an overview of the doctoral programme with regard to the nature of the mission, faculty, doctoral students, curriculum, programme administration and infrastructure, availability of institutional resources and evaluation of the programme. Section 2 (7 items) focused on doctoral programmes that offer research doctorates without coursework requirements; and Section 3 gathered biographical data.

#### Trustworthiness

The surveys for faculty, graduates and students had seven sections evaluating the quality of doctoral education and asked about the mission of the institution (2 items), the doctoral programme (17 items), faculty (12 items), resources (9 items), evaluation (8 items), doctoral programmes that offer research doctorates without coursework requirements (6 items) and biographical information. For Sections 1–6 of the survey, the items are answered on a four-point Likert Scale (strongly agree to strongly disagree). Since the items in the survey were formative constructs, conventional psychometric assessment procedures for reflective constructs could not be employed to assess the validity of this survey (Roy et  al. 2012), thus variance inflation factor (VIF) was employed. VIF values ranged from 1.8 to 3.5 for programme, 2.0 to 3.9 for faculty, 1.9 to 3.9 for resources and 1.3 to 3.8 for evaluation domain, showing that all formative domains did not have multicollinearity problems and met the requirement of indicator reliability (VIF > 10) (Diamantopoulos & Sigauw 2006). Hence, all four domains were considered to be appropriate measures of quality domains.

#### Data treatment

Data were captured using the computer program EPIDATA 3.1 (2008), which allows double entry verification, then analysed using IBM SPSS Statistics for Windows, Version 23.0. (IBM Corp., Armonk, NY 2014), which provided descriptive statistics, statistical significance and measures of effect sizes. More emphasis is placed on effect sizes (practical significance) which are given by Cohen's d-values, which is the standardised difference between the means of two populations, that is, the difference between the two means divided by the estimate for standard deviation (Ellis & Steyn 2003). Cohen (1988) gives the following guidelines for the interpretation of effect sizes: small effect, *d* = 0.2; medium effect, *d* = 0.5; and large effect, *d* = 0.8.

## Results

### Characteristics of the participants

From 15 doctoral nursing programmes, 12 deans out of 15 (80%), 26 faculty out of 50 (52%), 24 graduates out of 43 (56%) and 63 students out of 106 (59%) returned valid surveys. The average age of deans and doctorally-prepared faculty in SA is lower than that of the USA (see Table 1), where the average age of faculty holding the ranks of professor, associate professor and assistant professor is 60.5, 57.1 and 51.5 years (AACN 2013). However, reflecting on the average age of doctoral graduates and students, from whom we hope to recruit for academia, most are almost the same age as those currently in academe and their average age is higher than the national average age of doctorates and doctoral graduates in the USA, at 40 and 46.2 years, respectively (Berlin & Sechrist 2002). The majority of doctoral graduates and students are already employed in academe; this may be because of the fact that nurses with doctorates are not readily recognised in the SA marketplace. The average time that deans, faculty and doctoral graduates took to complete their doctoral degrees were 3.7, 3.8 and 3.6 years, respectively, whilst student were registered for 3.2 years on average. The completion rates are well within the permitted time range of two to five years for full-time study and three to seven years for part-time study, comparing favourably with overall health sciences completion rates of 4.5 years (CHE 2009).

**TABLE 1:** Characteristics of participants.

Variables	Deans *n* (%)	Faculty *n* (%)	Graduates *n* (%)	Students *n* (%)
Age [years, means (SD)]	53.9 (7.7)	53 (5.5)	51.3 (7.7)	46.3 (8.2)
**Gender**				
Male	-	-	-	3 (4.8)
Female	12 (100)	26 (100)	24 (100)	60 (95.2)
**Employment**				
Full-time academic	12 (100)	26 (100)	16 (66.7)	33 (52.4)
Part-time academic	-	-	2 (8.3)	2 (3.2)
Hospital	-	-	3 (12.6)	6 (9.8)
Other	-	-	3 (12.6)	18 (25.8)
Unemployed	-	-	-	4 (6.4)
**Number of years to complete doctoral degree**				
2–3	6 (60)	10 (38.4)	13 (56.5)	-
4–5	4 (40)	14 (53.8)	7 (30.5)	-
6–7	-	2 (7.7)	3 (13.1)	-
**Number of years registered for doctoral degree**				
1–2	-	-	-	22 (37.9)
3–4	-	-	-	22 (37.9)
5–6	-	-	-	14 (24.1)

### Mission of the institution

In the analysis, responses of ‘strongly agree’ and ‘agree’ were considered positive, whilst those of ‘disagree’ and ‘strongly disagree’ were considered to be negative. Items in this section had no significant difference with regard to the perceptions of the different participant groups. ‘The emphasis of the programme content is consistent with the mission of the university and the discipline of nursing’ was rated lowest by all the groups. Deans were asked ‘How closely do the vision, goals, mission, and objectives of the nursing doctoral programme align with those of your institution?’ and only 58.4% answered in the affirmative.

### Quality of the faculty

Table 2 presents significant differences amongst groups of participants in response to 5 of the 13 items evaluating the faculty: Items 2.1, 2.3, 2.4, 2.5 and 2.13. As to Item 2.1, ‘Faculty members meet the requirements of the institution for graduate research and doctoral education’, responses were highest for graduates (100%), followed by students (93.2%) and faculty (88.5%). Over 70% of graduates and students responded positively to Item 2.3, that faculty have evidence of extramural support for their research and for their success in obtaining funding support for their students, whilst only 50% of faculty had an affirmative response. As to Item 2.4, that faculty have sufficient evidence of scholarship and have published in peer-reviewed journals, close to 90% of students and 85% of graduates responded positively, but fewer faculty (76.9%) responded the same way. Faculties were requested to indicate the number of papers published in peer-reviewed journals in the previous three years, of whom 30.8% had published fewer than three articles. As to Item 2.5, ‘Faculty have teaching experience in nursing education prior to working with doctoral students’, the percentage of strongly agree responses was highest for students (70.8%), followed by graduates (59.7%) and faculty (53.9%). For Item 2.13, rating the overall quality of teaching by faculty, graduates responded most positively (87.5%), followed by students (80.7%) and faculty (78.2%). All other items in the table had little or no difference in the perceptions of different participant groups.

**TABLE 2:** Quality of the faculty: Faculty, graduate and student perspectives.

Item number	Quality of the faculty	Faculty			Graduates			Students			Stat Sig	Effect sizes	
		***n***	**M**	**SD**	***n***	**M**	**SD**	***n***	**M**	**SD**	***p***	**Faculty (*d*)**	**Graduates (*d*)**
2.1	Faculty members meet the requirements of the institution for graduate research and doctoral education.	21	3.2	0.7	23	3.6	0.5	59	3.4	0.7	.157	0.55	-
												0.29	0.26
2.2	Faculty members have expertise in the subject area appropriate for student learning.	21	3.4	0.6	23	3.5	0.5	58	3.3	0.7	.518	0.16	-
												0.12	0.26
2.3	Faculty members have evidence of extramural support for their research and for their success in obtaining funding support for their students, such as fellowships or bursaries.	21	2.6	0.9	23	2.9	0.9	61	3.0	0.9	.236	0.27	-
												0.41	0.15
2.4	Faculty members have sufficient evidence of scholarship, and have published in peer-reviewed journals.	21	3.0	0.7	24	3.3	0.8	60	3.3	0.7	.176	0.50	-
												0.40	0.11
2.5	Faculty members have teaching experience in nursing education prior to working with doctoral students.	21	3.5	0.5	24	3.7	0.5	62	3.6	0.5	.291	0.45	-
												0.24	0.23
2.6	Faculty members provide students with diverse and challenging learning experiences (e.g., social, ethical, cultural, economic, and political issues related to nursing, health care, and research).	20	2.8	01.0	24	3.0	0.8	62	3.1	0.8	.358	0.21	-
												0.31	0.12
2.7	Faculty members have been certified in nursing specialties and hold membership in professional organisations/societies.	21	3.4	0.7	24	3.5	0.7	63	3.6	0.5	.435	0.16	-
												0.26	0.11
2.8	Faculty members demonstrate fulfilment of diverse faculty responsibilities and roles, including teaching, research, service, and mentoring.	20	3.3	0.8	24	3.3	0.6	63	3.4	0.6	.610	0.05	-
												0.19	0.17
2.9	Faculty members mentor and assist students to understand the value of programmes of research and scholarship.	21	3.1	0.8	23	3.1	0.8	62	3.2	0.8	.867	0.04	-
												0.12	0.08
2.10	Faculty members utilise resources within the institution and broader community to support programme goals.	21	3.0	0.8	24	3.1	0.9	62	3.2	0.7	.418	0.20	-
												0.32	0.10
2.11	Faculty members devote significant time to students’ dissertation/thesis research.	21	3.1	0.9	24	3.1	0.8	61	3.3	0.8	.663	0.07	-
												0.12	0.20
2.12	Faculty members give timely feedback on students’ research.	20	3.2	0.8	24	3.3	0.8	61	3.2	0.7	.685	0.17	-
												0.00	0.19
2.13	How would you rate the overall quality of teaching by faculty in your doctoral programme?	23	3.0	0.8	24	3.4	0.7	62	3.1	0.7	.139	0.55	-
												0.18	0.38

Stat sig, statistical significance; SD, standard deviation.

### Quality of students

According to the deans, an average of 6.1 students (range 0–16; SD 4.7) are being admitted per nursing doctoral programme per annum, with an average of 0.9 students (range 0–3; SD 1.3) graduating per nursing doctoral programme per annum. With regard to academic outputs, 45.8% of graduates and 66.7% of students had neither authored nor co-authored articles in peer-reviewed articles and 33.3% of graduates and 61.9% of students had made no presentations at national conferences.

### Quality of curriculum

Table 3 indicates that responses to five of the nine items differed significantly between the groups of participants and their evaluation of the curriculum (goal and content): Items 3.1, 3.3, 3.7, 3.8 and 3.9. As to Item 3.1, ‘There is a clear emphasis on nursing science and research training in the programme content’, responses were highest for graduates (95.4%), followed by students (90.2%) and faculty (76%). Students (90.3%) and graduates (82.6%) rated positively the fact that the programme content includes core information and other relevant information appropriate for a doctoral degree in nursing, but fewer faculty (70.8%) responded the same way. For Item 3.7, rating the programme content of the doctoral programme, graduates responded most favourably (87%), followed by faculty (65.3%) and then students (64.4%). Whilst 91.7% of the graduates responded positively to Item 3.8, ‘How would you rate the intellectual liveliness of your programme?’, fewer students (77.4%) and faculty (64%) agreed. In rating the benefit of the overall intellectual environment (Item 3.9), graduates rated this item the highest (100%), followed by students (93.6%) and then faculty (79.1%). All other items in the table had little or no difference in the perceptions of different participant groups.

**TABLE 3:** Quality of the curriculum (goal and content): Faculty, graduate and student perspectives.

Item number	Quality of the curriculum (goal and content)	Faculty			Graduates			Students			Stat Sig	Effect sizes	
		***n***	**M**	**SD**	***n***	**M**	**SD**	***n***	**M**	**SD**	***p***	**Faculty (*d*)**	**Graduates (*d*)**
3.1	There is a clear emphasis on nursing science and research training in the programme content.	24	3.1	0.9	22	3.5	0.6	61	3.4	0.7	.235	0.37	-
												0.28	0.12
3.2	Faculty research expertise areas (e.g., nursing ethics, women's health, biobehavioural science, genetic nursing etc.) are presented in the programme content.	23	2.9	0.8	23	3.1	1.0	62	2.6	0.8	.404	0.26	-
												0.02	0.27
3.3	The programme content includes core information (e.g., theory development, research methodologies for qualitative and quantitative research, ethical considerations in research, dissertation/thesis seminars, etc.) and other relevant information (e.g., leadership, policy, etc.) appropriate for a doctoral degree in nursing.	23	3.0	0.9	23	3.4	0.9	62	3.4	0.7	.085	0.38	-
												0.48	0.10
3.4	All students receive formal training in ethics and the protection of human/animal subjects in research.	24	2.9	0.8	22	2.9	1.0	61	2.8	0.7	.787	0.03	-
												0.11	0.12
3.5	Programme descriptions are written and available to students and faculty in detail.	24	2.9	0.9	22	3.1	0.8	61	3.1	0.8	.518	0.24	-
												0.25	0.01
3.6	The programme includes interdisciplinary dissertation/thesis research seminars and interdisciplinary courses in addition to seminars.	24	2.9	1.1	23	3.0	0.9	63	3.3	0.8	.190-	0.04	-
												0.31	0.34
3.7	How would you rate the programme content of your PhD/doctoral programme?	20	3.1	1.1	23	3.3	0.7	59	2.8	0.8	0.37	0.24	-
												0.26	0.65
3.8	How would you rate the intellectual liveliness of your programme?	25	2.9	0.9	24	3.3	0.6	62	3.0	0.7	2.281	0.47	-
												0.10	0.46
3.9	Considering the overall intellectual environment of your school/ department/ division and university, how much do you think you have benefited from it?	24	3.2	0.9	24	3.8	0.4	63	3.5	0.6	4.831	0.66	-
												0.32	0.48

Stat sig, statistical significance; SD, standard deviation.

### Quality of supervision

Faculty reported that on average they were supervising 3.4 (SD 6.8) doctoral students, ranging from 0–13, and supervising 9.2 master's students, ranging from 3–28. Responses to one of the five items evaluating the curriculum (supervision) differed significantly between the groups of participants, namely, Item 4.5 (see Table 4). This item rating the overall quality of supervision had the most variance between groups, with 91.7% of graduates responding positively, followed by 80.9% students, 75% deans and 68% faculty.

**TABLE 4:** Quality of the curriculum (supervision): Deans, faculty, graduate and student perspectives.

**Item number**	**Quality of the curriculum (Supervision)**	**Deans**			**Faculty**			**Graduates**			**Students**			**Stat Sig**	**Effect sizes**		
		***n***	**M**	**SD**	***n***	**M**	**SD**	***n***	**M**	**SD**	***n***	**M**	**SD**	***p***	**Deans (*d*)**	**Faculty (*d*)**	**Graduates (*d*)**
4.1	Does your institution have well-developed systems to foster quality research including consultation on grant proposal and analysis of data?	12	2.6	0.9	24	2.5	1.1	24	2.7	0.9	61	2.6	0.8	.886	0.08	-	-
															0.14	0.19	-
															0.04	0.11	0.09
4.2	The emphasis of the supervision is consistent with the mission of the university and the discipline of nursing.	12	3.3	0.6	24	3.0	0.9	23	3.3	0.8	62	3.0	0.8	.390	0.27	-	-
															0.07	0.33	-
															0.31	0.00	0.37
4.3	Emphasis is on nursing science and research training through supervision.	12	3.1	0.8	25	3.0	0.9	23	3.3	0.8	63	3.0	0.7	.396	0.09	-	-
															0.28	0.33	-
															0.13	0.02	0.42
4.4	The supervision includes areas appropriate for a doctorate degree in nursing (e.g., theory development, research methodologies for quantitative and qualitative research, ethical consideration in research, dissertation/thesis seminars, etc.).	12	3.4	0.8	25	3.3	0.8	23	3.3	0.9	63	3.2	0.8	.800	0.17	-	-
															0.12	0.03	-
															0.29	0.11	0.12
4.5	How would you rate the quality of supervision in your doctoral programme?	12	2.8	0.5	25	3.0	0.8	24	3.3	0.6	63	3.2	0.8	.103	0.27	-	-
															0.92	0.47	-
															0.52	0.26	0.20

Stat sig, statistical significance; SD, standard deviation.

### Quality of administration and infrastructure

Items in this section had no significant difference with regard to the perceptions of the different participant groups (see Table 5).

**TABLE 5:** Quality of administration and infrastructure: Faculty, graduate and student perspectives.

**Item number**	**Quality of administration and infrastructure**	**Faculty**			**Graduates**			**Students**			**Stat Sig**	**Effect sizes**	
		***n***	**M**	**SD**	***n***	**M**	**SD**	***n***	**M**	**SD**	***p***	**Faculty (*d*)**	**Graduates (*d*)**
5.1	The institution values, supports, and provides rewards to students for their research and scholarly activity.	24	3.0	1.0	22	3.1	0.9	61	3.3	0.8	.292	0.05	-
												0.28	0.26
5.2	The institution has a well-developed system to foster quality research and scholarly activities.	22	3.0	0.8	23	3.2	0.7	61	3.2	0.8	.359	0.28	-
												0.34	0.07
5.3	The environment is supportive of students’ learning.	23	3.0	1.0	23	3.2	0.6	63	3.2	0.7	.364	0.17	-
												0.14	0.04
5.4	The programme has a process in place that fosters socialisation of students to doctoral education, and facilitates interaction amongst students, and between faculty and students.	24	2.6	1.1	22	2.7	0.8	61	2.8	0.9	.310	0.09	-
												0.16	0.09
5.5	There are administration systems in place to ensure that faculty carry out regular and appropriate supervision of students’ progress.	24	2.8	0.8	23	2.8	0.7	61	2.9	0.8	.210	0.01	-
												0.11	0.13
5.6	Sufficient materials and information are available for students (e.g., financial support, scholarships, grants, and resources).	24	2.8	1.1	23	3.0	0.9	62	3.0	0.9	.377	0.19	-
												0.17	0.02
5.7	Sufficient information about careers is available.	24	2.6	1.1	22	2.4	0.8	60	2.7	0.8	.310	0.25	-
												0.07	0.41
5.8	Faculty provide recommendation letters when needed and seek job opportunities for students.	24	2.8	0.9	22	2.9	0.8	59	2.7	0.8	.763	0.08	-
												0.09	0.18

Stat sig, statistical significance; SD, standard deviation.

### Quality of resources

Responses to 4 of the 10 items evaluating resources differed significantly between the groups of participants: Item 6.2, 6.3, 6.5 and 6.10 (see Table 6). Whilst 65.2% of students responded that ‘Number of technical and support staff is sufficient to support doctoral students’ (Item 6.2), only 60.6% of graduates and 36% of faculty made an affirmative response. As to Item 6.3, ‘Research infrastructure is appropriate for facilitating research and education’, responses were highest for students (72.9%), followed by graduates (69.6%) and faculty (56%). Close to 70% of students and faculty responded positively to Item 6.5, that advanced information technology is available for research and education off-site, if offered, but fewer (56.5%) of graduates responded the same way. For Item 6.10, regarding the availability of various sources of funding for student research, students responded most positively (62.7%), followed by graduates (62.5%) and faculty (41.7%); this item was also rated lowest by all participant groups.

**TABLE 6:** Quality of resources: Faculty, graduate and student participants.

**Item number**	**Quality of resources**	**Faculty**			**Graduates**			**Students**			**Stat Sig**	**Effect sizes**	
		***n***	**M**	**SD**	***n***	**M**	**SD**	***n***	**M**	**SD**	***p***	**Faculty (*d*)**	**Graduates (*d*)**
6.1	There are sufficient numbers of faculty to facilitate learning.	24	2.6	0.9	22	2.7	1.0	61	2.7	0.8	.281	0.10	-
												0.17	0.06
6.2	Number of technical and support staff is sufficient to support doctoral students.	25	2.2	0.8	23	2.8	0.9	61	2.6	0.8	.021	0.67	-
												0.54	0.20
6.3	Research infrastructure is appropriate for facilitating research and education.	25	2.6	0.9	23	3.0	1.0	59	2.9	0.8	.180	0.41	-
												0.41	0.04
6.4	Advanced computing facilities with Internet access are in place.	25	3.3	0.8	23	3.1	1.0	62	3.3	0.7	.572	0.19	-
												0.00	0.19
6.5	Advanced information technology is available for research and education at off-sites, if offered.	25	3.1	0.9	23	2.7	1.1	61	2.9	0.9	.277	0.38	-
												0.28	0.16
6.6	Library has sufficient holdings, search engines, and databases.	25	3.6	0.7	22	3.5	0.8	63	3.3	0.7	.407	0.13	-
												0.31	0.15
6.7	School/department/division building provides sufficient space for student activities (e.g., seminar, offices, student lounge).	25	2.5	1.0	23	2.7	1.0	62	2.8	0.8	.427	0.26	-
												0.28	0.02
6.8	School/department/division is equipped with sufficient resources for teaching and research (e.g., computers, photocopiers, teleconference capability).	25	2.8	0.9	22	2.7	1.1	63	2.9	0.8	.563	0.15	-
												0.09	0.22
6.9	School/department/division has relevant and ancillary facilities for education, training and research (e.g., affiliated hospitals, community health agencies).	23	3.0	0.9	23	3.0	1.0	59	3.1	0.7	.943	0.05	-
												0.03	0.07
6.10	The school/department/division has various sources of funding for student research.	24	2.4	1.0	24	2.8	0.9	59	2.7	0.8	.257	0.41	-
												0.28	0.14

Stat sig, statistical significance; SD, standard deviation.

### Quality of evaluation

Table 7 shows that the responses to one of the five items evaluating programme evaluation differed significantly between the groups of participants, namely, Item 7.4. Whilst 75% of graduates responded that ‘The school provides comprehensive data in order to determine patterns and trends of nursing doctoral education and recommend future directions at regular intervals’, only 71% of students and 56.3% of faculty made an affirmative response.

**TABLE 7:** Quality of evaluation: Faculty, graduate and student perspectives.

**Item number**	**Quality of evaluation**	**Faculty**			**Graduates**			**Students**			**Stat Sig**	**Effect sizes**	
		***n***	**M**	**SD**	***n***	**M**	**SD**	***n***	**M**	**SD**	***p***	**Faculty (*d*)**	**Graduates (*d*)**
7.1	Programme evaluation systems adhere to ethical and procedural standards for formal programme evaluation (e.g., confidentiality).	16	3.3	0.6	12	3.5	0.5	41	3.4	0.6	.721	0.31	-
												0.12	0.17
7.2	Students and graduates have been involved in programme evaluation activities.	16	2.9	0.9	12	2.8	1.1	40	2.7	0.9	.569	0.09	-
												0.31	0.16
7.3	Programme evaluation is systematic, ongoing, and comprehensive and focuses on the institutions’ and programme's specific mission.	16	2.9	1.0	12	3.3	0.6	37	3.0	0.9	.613	0.31	-
												0.06	0.28
7.4	School/department/division provides comprehensive data in order to determine patterns and trends of nursing doctoral education and recommend future directions at regular intervals.	16	2.6	0.9	12	2.9	0.9	38	2.8	0.8	.503	0.39	-
												0.28	0.11
7.5	Regular feedback is provided to programme faculty, administrators, and external constituents.	16	2.9	1.1	12	2.8	0.8	37	2.9	0.8	.912	0.11	-
												0.01	0.15

Stat sig, statistical significance; SD, standard deviation.

## Discussion

### Current status of nursing doctoral education in South Africa

The major global problem that has been identified is the shortage of faculty with doctorates in nursing. In SA, this shows little prospect to improve in the short term, when one considers that 70% of deans and 72% of faculty are more than 50 years old. Of these, 70% are due to retire in the next nine to 14 years. Furthermore, with regard to the future of nursing academe, doctoral graduates and students are also an ageing population, the average graduate having an age of 51 years and the students, 46 years. Also worth noting is that 75% of the graduates and 56% of the students are already absorbed in academe, which means that there is a limited pool from which retiring faculty can be replaced; and higher compensation in clinical and private-sector settings is luring current and potential nurse educators away from teaching. Further contributing to this problem is that there are some nursing schools that had admitted no students into the doctoral programme in the prior three years and furthermore, during that period, had conferred no nursing doctorates. The national graduation rate for doctorates in nursing is less than 20% per annum, causing a pile-up effect of over 80% of doctoral students per annum.

There are many reasons for the low graduation rate and the pile-up effect. Key amongst them is a shortage of faculty and the relative lack of supervision experience, which results in their having a high burden of supervision, with each faculty member having an average of 9.2 master's students and 3.4 doctoral students. Also, students enter the doctoral programme with little or no research training and 87% of them study part-time, having to balance full-time employment with their studies. Furthermore, as uncovered in our research, 55% of deans and 68% of faculty have recently obtained their doctorates. Nevertheless, they had been pushed into leadership and management positions with little or no mentoring because of a dearth of senior role models and the shortage of faculty.

The high workload and, in many cases, lack of relevant experience directly affect the research programme and careers of faculty, as over 65% of them do not have the opportunity to attend training programmes allowing them to be mentored in launching and advancing their further scholarly development. The nursing discipline as a whole is held back in these conditions: only five academics have a research rating in the nursing field by the country's National Research Foundation (NRF 2015); 73% of nursing academics publish less than the institutional requirement of one peer-reviewed article equivalent a year; 66% of faculty have not accessed funding from externally-reviewed sources; and 11.5% had not presented research findings at national conferences in the previous three years.

Their problems filter down to the student level. Those registered for doctorates are supervised by newly-qualified and often inexperienced academics. This situation is reflected in disappointing outputs, with over a quarter of graduates and half of doctoral students never having presented their work at conferences. Similarly, almost half (45.8%) of graduates and more than two-thirds (66.7%) of students have never published peer-reviewed articles.

Of further note is that less than 40% of nursing schools evaluate their nursing doctoral programmes regularly, with the result that they have no baseline data from which to identify threats to quality, or even to design and implement strategies to minimise the threats.

### Difference amongst evaluators on items

The quality of faculty, followed by the quality of the curriculum and the quality of resources, were the criteria in which the greatest number of items yielded significantly different responses amongst the participant groups. Significant differences amongst groups were reported in 5 of the 13 items related to the quality of faculty, in which the graduates identified more strengths than either the students or faculty. Faculty rated all 5 of these items most negatively and also gave the lowest rating for 12 of the 13 items related to faculty. The fact that faculty rated these criteria most negatively may point to the dearth of senior role models from whom faculty can learn, as well as inadequate opportunities offered for formal training to advance their professional development. With regard to the quality of the curriculum, significant differences were reported in five of the nine items: graduates rated the items most positively, followed by students and faculty. Faculty rated four of the five items most negatively, with students rating the overall programme content (Item 3.7) lowest. The fact that students rated the programme content most negatively may result from the fact that the doctoral programme in SA has no prescribed coursework, causing the student to feel that the programme does not prepare the student sufficiently to conduct doctoral studies. Significant differences amongst groups were reported in four of the 10 items related to the quality of resources, in which the students identified more strengths than either the graduates or faculty. Faculty rated three of the four items most negatively, with graduates rating lowest the fact that advanced information technology is available for research and education off-site, if offered (Item 6.5).

### Current weaknesses of nursing doctoral education

Here we highlight the items identified by participating groups in each of the quality criteria as needing the most improvement. With regard to the quality of faculty, all groups gave a poor rating to Item 2.1, regarding faculty having evidence of extramural support for their research. In our study, faculty were asked whether they had received extramural funding from externally-reviewed sources in the previous five years: 56% had received no extramural funding, 20% had received national funding and 24% had received international funding. Furthermore, only 26.9% of faculty indicated that they could support doctoral students financially from their research projects. In light of this result, it was not surprising that students reported having to depend largely on their own personal savings and earnings to pay for doctoral education: only half (50.8%) receive a grant or bursary from the institution, of which less than half (41.9%) received full tuition remission. With regard to the curriculum, the item rated most poorly by the groups was Item 3.4, which related to receiving no formal training in ethics. This is a cause for concern, as ethics is the foundation for good scientific research. Regarding supervision, Item 4.1 concerning the availability of a well-developed system to foster quality research, including consultation on grant proposal and analysis of data, was scored most poorly by all the groups. This highlights the need for both providers and receivers to have access to expert consultation on grant proposal and data analysis. Interestingly, Item 5.7 regarding sufficient information about careers was highlighted as a weakness by all groups in the criteria of administration and infrastructure. With regard to quality of resources, three items were rated poorly in close succession. Item 6.2, the lack of technical and support staff, came first; followed closely by Item 6.10, the lack of sources of funding for student research, and Item 6.1, the lack of sufficient faculty to facilitate learning. Finally, with regard to evaluation, Item 7.4 concerning the nursing school providing comprehensive data to determine patterns and trends of nursing doctoral education and recommend future directions at regular intervals, scored most poorly and is verified by the fact that as many as 40% of nursing schools do not evaluate their doctoral programmes. This result alone justifies the need for this study, which for the first time has evaluated the quality of doctoral nursing education in SA.

## Limitations of the study

The length of the questionnaire was one of the most important factors contributing to a lower than expected response rate. However, this study was part of an international collaborative study, therefore we did not have the prerogative to adjust the surveys without compromising the international study. Furthermore, although the entire population was approached to participate in the study, only half of graduates and students did so; however, a 50% response rate is high in general survey study and both deans and faculty were well represented.

## Recommendations

First and foremost, we recommend that nursing schools regularly evaluate the quality of their doctoral programmes, so as to provide feedback to faculty, administrators and customers (internal and external) and also to provide all nursing schools with the opportunity to perform both national and international benchmarking. Secondly, there must be an expansion of the scholarship, career development and innovation capacity of faculty by encouraging them to pursue funding actively from externally-reviewed sources, apply for research rating and improve scientific outputs; by providing training to develop staff; by increasing capacity for supervision and mentorship; and by developing and focusing the nursing schools’ research strengths and priorities. Finally, we recommend that effort be invested to increase the human resource base and age profile of faculty by raising government and public awareness of the shortage of faculty with nursing doctorates, increasing the pool of potential faculty at nursing schools, incorporating a scarce skill allowance and creating positive work environments for them.

## Conclusion

Our research is the first to present the current status of nursing doctoral education in SA. The study further made use of providers and receivers of doctoral education as evaluators; the significant differences reported amongst these groups exemplifies the need to understand the quality of nursing doctoral education from these different perspectives. Overall, the graduates scored their programme quality most positively of all the groups and faculty scored it most negatively. Areas that need further study are the possibility of introducing more coursework in the doctoral programme and increasing resources to support doctoral education.
